# Coenzyme Q10, Ageing and the Nervous System: An Overview

**DOI:** 10.3390/antiox11010002

**Published:** 2021-12-21

**Authors:** David Mantle, Robert A. Heaton, Iain P. Hargreaves

**Affiliations:** 1 Pharma Nord (UK) Ltd., Morpeth NE61 2DB, UK; 2 School of Pharmacy and Biomolecular Sciences, Liverpool John Moores University, Liverpool L3 3AF, UK; r.heaton@2013.lmju.ac.uk (R.A.H.); i.hargreaves@ucl.ac.uk (I.P.H.)

**Keywords:** coenzyme Q10, Alzheimer’s disease, Parkinson’s disease, amyotrophic lateral sclerosis, stroke, multiple system atrophy, ophthalmic disorders, neurodegenerative disorders, ageing

## Abstract

The ageing brain is characterised by changes at the physical, histological, biochemical and physiological levels. This ageing process is associated with an increased risk of developing a number of neurological disorders, notably Alzheimer’s disease and Parkinson’s disease. There is evidence that mitochondrial dysfunction and oxidative stress play a key role in the pathogenesis of such disorders. In this article, we review the potential therapeutic role in these age-related neurological disorders of supplementary coenzyme Q10, a vitamin-like substance of vital importance for normal mitochondrial function and as an antioxidant. This review is concerned primarily with studies in humans rather than in vitro studies or studies in animal models of neurological disease. In particular, the reasons why the outcomes of clinical trials supplementing coenzyme Q10 in these neurological disorders is discussed.

## 1. Introduction

The ageing brain is characterised by changes at the physical, histological, biochemical and physiological levels. At the physical level, the volume of the brain shrinks with increasing age; this process appears to begin after the age of forty and is particularly marked after the age of seventy [1]. Different areas of the brain may be differentially affected by this shrinkage process, with the prefrontal cortex (associated with complex cognitive behaviour) being particularly susceptible [2]. At the histological level, the volumes of both white and grey matter are reduced with increasing age [3]. Whilst the number of neuronal cells may not be significantly reduced during normal ageing, neurons may undergo shrinkage, with a reduction in axonal length, degeneration of the myelin sheath and reduced numbers of synaptic contacts [4]. Similarly, whilst numbers of glial cells may not be significantly reduced with ageing, the supportive role of astrocytes, oligodenrocytes and microglia may compromised, rendering neurons more susceptible to damage [5]. At the biochemical level, ageing is characterised by reductions in the levels of a number of neurotransmitters, including acetylcholine, dopamine and serotonin [6]. In addition, there is evidence of mitochondrial dysfunction in brain tissue with increasing age, both with regard to energy production and to increased generation of free radical species [7]. Cellular damage resulting from free radical-induced oxidative stress has been implicated in both normal ageing and in various neurodegenerative disorders [8]. At the physiological level, cognitive function is affected by ageing, particularly the speed of information processing and memory [9]. In addition, the blood supply to the ageing brain may be impaired [10] and there is evidence for partial breakdown of the blood–brain barrier [11]. This review is concerned primarily with studies in humans rather than in vitro studies or studies in animal models of neurological disease. In particular, the reasons why the outcomes of clinical trials supplementing coenzyme Q10 in these neurological disorders is discussed.

## 2. Neurological Disorders and Ageing

Ageing is associated with an increased risk of developing a number of neurological disorders, including dementia (Alzheimer’s disease, vascular dementia), Parkinson’s disease, amyotrophic lateral sclerosis (ALS) and stroke. Thus, the risk of developing the progressive neurodegenerative disorder Alzheimer’s disease (which accounts for approximately 75% of all dementia cases) increases from approximately 6% at age 65 to 36% at age 85 [12]. Similarly, the risk of developing Parkinson’s disease is less than 1% at age 60 and 5% at age 85 [13]. ALS is relatively uncommon before the age of forty but increases exponentially thereafter; the age of onset is typically in the range 55–75 years, with a peak incidence in those aged 70–80 years [14]. With regard to strokes, the risk doubles with every decade after 50 years of age, affecting approximately 20% of the population at age 75 years [15]. Such age-related neurological disorders represent a burden to patients and their carers and to health and social services. Thus, in the UK, patients with these neurological disorders are rated to have the lowest health-related quality of life, whilst these conditions consume a significant proportion of the NHS and social services budgets (www.england.nhs.neurologicalconditions.uk, accessed on 11 November 2021). For example, the annual cost of dementia to the NHS is approximately 4 billion GBP, with corresponding social care costs of approximately 10 billion GBP. In addition, the annual cost of strokes to the NHS is approximately 3 billion GBP and Parkinson’s disease more than 200 million GBP, respectively. Other neurological disorders with age-associated increased risk include multiple system atrophy and some forms of ataxia, as well as sensory disorders, such as glaucoma, macular degeneration and presbycusis (hearing loss).

## 3. Coenzyme Q10 and Ageing

**Figure 1:**
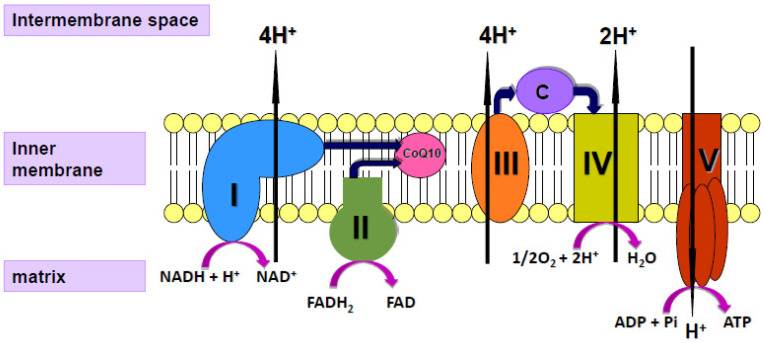
Diagram of the mitochondrial respiratory chain (MRC) showing the enzyme complexes I V and the electron carriers’ coenzyme Q10 (CoQ10), cytochrome c (C) and protons (H^+^).

Coenzyme Q10 (CoQ10) is a vitamin-like substance synthesised in most tissues of the human body. CoQ10 has a key role in the process of cellular energy supply via oxidative phosphorylation within mitochondria, shuttling electrons from complexes I and II to complex III of the mitochondrial respiratory chain (MRC; Figure 1); CoQ10 is also a powerful lipid-soluble antioxidant, protecting cell membranes from free radical-induced oxidative damage [16]. In addition to its role in mitochondrial function, CoQ10 is present in other subcellular organelles, including lysosomes, peroxisomes, Golgi apparatus and endoplasmic reticulum. As well as providing antioxidant protection for these organelle membranes from oxidative stress, CoQ10 has a role in maintaining the intralysosomal pH [17]. CoQ10 has been shown to directly affect the expression of a number of genes, including those involved in the inflammatory process [18]. Although a small amount of CoQ10 (of the order of 5 mg/day) is obtained from the normal diet [19], most of the body’s daily CoQ10 requirement is derived from endogenous synthesis. The daily requirement for CoQ10 is not known with certainty but has been estimated to be approximately 500 mg/day, based on a total body pool of 2000 mg and an average tissue turnover time of 4 days [19].

In humans, CoQ10 levels are typically measured in blood samples, although there is some discussion as to how accurately blood CoQ10 levels reflect those within other tissues; CoQ10 levels may, therefore, be measured in muscle biopsy specimens although less frequently [20]. In view of the preponderance of neurological dysfunction associated with a deficit in CoQ10 status, it would seem appropriate to determine patient cerebral CoQ10 status. Cerebral spinal fluid (CSF) has been suggested as an appropriate surrogate to use in order to evaluate cerebral CoQ10 status [20]. In view of the low levels of CoQ10 reported in this matrix, only highly sensitive analytical techniques, such as HPLC-ED (electrochemical detection) and tandem mass spectrometry, would appear appropriate for this analysis due to their low detection limits [20]. A tentative reference range of 5.7–9.0 nM has been established for CSF CoQ10 status in humans [21].

Relatively little data are available in the published literature regarding changes in CoQ10 levels in various human tissues as a function of age. However, in general terms, optimum CoQ10 production occurs around 25 years of age, with a subsequent age-related decline that may vary in different tissues [22]. For example, in heart tissue, the production of CoQ10 at age 65 is approximately half of that at age 25 years. At present, there is no comparable data for changes in CoQ10 levels in the human brain as a function of normal ageing. CoQ10 is of relevance to normal neurological function and the risk of developing neurological disorders on the basis of the high-energy requirement (equivalent to 20% of total body energy consumption) for normal brain function and the implication of mitochondrial dysfunction and free radical-induced oxidative stress in the pathogenesis of a number of neurological disorders. Coenzyme Q10 is present in all areas of the brain [23]; both neurons and glia contain CoQ10, and supplemental CoQ10 has been shown to restore depleted levels in cultured human neuronal cells [24]. The age-related changes in neurological function and increased risk of developing neurological disorders are paralleled by the age-related depletion of tissue CoQ10 levels. The question, therefore, arises as to whether supplementation with CoQ10 could address these issues, and this is discussed in the following sections. To date, there have been no randomised controlled trials to assess the benefit of CoQ10 supplementation on cognitive function in the normal elderly. However, a 90-day randomised controlled trial has now been registered with the Australian and New Zealand Clinical Trials Registry (ANZCTRN12618001841268) to assess the ability of ubiquinol supplementation to ameliorate cognitive decline in healthy male and female volunteers aged 60 and above. This study will be the first of its kind to investigate the potential of ubiquinol treatment to arrest the age-related cognitive decline, and its results are awaited with anticipation.

## 4. Coenzyme Q10 and Neurological Disorders

**Alzheimer’s disease:** Alzheimer’s disease (AD) is a progressive neurodegenerative disorder characterised by initial memory impairment and cognitive decline. AD generally presents after the age of 65 years, although there are cases of AD that present before this age, referred to as early-onset AD [25]. AD is the most common form of dementia and is characterised by an accumulation of abnormal neuritic plaques composed of β-amyloid peptide, together with neurofibrillary tangles of misfolded tau protein, in the brain of AD patients [26,27]. The causes of AD have yet to be fully elucidated, with a myriad of genetic and environmental factors associated with the development of this disorder [28]. However, oxidative stress is thought to be an important factor in disease pathogenesis, reportedly preceding the appearance of neurofibrillary tangles and senile plaques, although the origin of the oxidative stress is uncertain [29]. In view of the potent antioxidant propensity of CoQ10, studies have been undertaken to assess the endogenous CoQ10 status of patients with AD. However, no evidence of a deficiency in the circulatory level of this isoprenoid has so far been reported [30,31]. Nonetheless, a population-based prospective cohort study by Yamagishi et al. [32] has suggested that serum CoQ10 levels may be a predictor rather than a biomarker of dementia. In vitro studies using human vein endothelial cells (HVEC) have indicated the potential therapeutic potential of CoQ10 supplementation in preventing the harmful effects of β-amyloid deposition in the early asymptomatic stages of AD, which may delay the progression of the neuropathology [33]. Pre-treatment of the HVEC with CoQ10 prevented β-amyloid-induced cellular toxicity and oxidative injury by inhibiting β-amyloid trafficking and accumulation in the mitochondria [33]. In a rat model of AD, an injection of β-amyloid was found to significantly increase the serum level of malondialdehyde, an end product of lipid peroxidation, and total oxidant levels. CoQ10 supplementation was found to significantly reverse these consequences and increase the total antioxidant capacity of the serum [34]. The results of this study have indicated the potential of CoQ10 to offer some protection against the oxidative stress associated with AD. However, in a randomised clinical trial in which 70 patients with mild to moderate AD were treated with CoQ10 (400 mg; three times/day) for 16 weeks, no clinical benefit or significant effect on the CSF biomarkers for AD (amyloid-beta and tau protein levels) were reported [35]. To date, no large clinical studies have assessed the cognitive effect of CoQ10 supplementation in AD; however, clinical studies with the CoQ10 analogue idebenone have reported modest cognitive and behavioural improvements in patients following supplementation [36,37]. There were, however, drop-out rates as high as 71% in these studies. A subsequent, one-year, multicentre, double-blind, placebo-controlled, randomised clinical trial of 536 AD patients found that idebenone treatment (120, 240 or 360 mg, three times a day) failed to slow cognitive decline in AD [38].

**Parkinson’s disease:** Parkinson’s disease (PD) is a chronic and progressive neurodegenerative disorder characterised by proteinaceous intraneuronal Lewy body formation and striatal dopamine depletion in the substantia nigra of the mid-brain [39]. This disease, which is the second most common neurodegenerative disorder after AD, with an incidence of approximately 13.4 per 100,000, is most commonly diagnosed in people over the age of 60, although 4% of cases present under the age of 50 [40]. Patients with PD commonly experience motor symptoms, such as bradykinesia, tremor, muscle stiffness (rigidity) and postural instability [41]. Whilst the preponderance of cases of PD are spontaneous or idiopathic, a genetic origin for this disease has also been reported in approximately 15% of patients [42]. Evidence for the involvement of MRC in the pathogenesis of PD emerged in the early 1980s with reports of Parkinsonian-like presentation of intravenous users of the synthetic drug, 1-methyl-4-phenyl-1,2,3,4-tetrahydropyridine (MPTP), which was also known as synthetic heroin [43]. MPTP is converted into the neurotoxin, 1-methyl-4-phenylpyridinium (MPP+) via a reaction catalysed by monoamine oxidase-B (MAO-B) enzyme. MPP+ was found to induce a Parkinsonian-like state in rats and primates by the inhibition of MRC complex I activity [43]. A subsequent study by Schapira et al. [44] reported evidence of decreased MRC complex I activity in post-mortem brain samples from PD patients. At present, the origin of the cerebral MRC complex I deficiency in PD has yet to be fully elucidated, although oxidative stress is thought to be an important contributory factor [45]. Together with evidence of impaired MRC complex I activity, a deficiency in cerebral CoQ10 status has also been reported in PD patients [23]. A decrease in CoQ10 status has also been reported in both the plasma and platelets of PD patients [46,47]. The cause of the decrease in CoQ10 status associated with PD is at present uncertain, although it is thought to be associated with the increase in oxidative stress associated with this condition, which may either increase the catabolism of CoQ10 or inhibit the enzymes involved in its biosynthesis [48]. Although the functional consequences of a CoQ10 deficiency in PD are at present unclear, a decrease in CoQ10 status would be expected to contribute to disease pathophysiology by compounding MRC function, as well as compromising cellular antioxidant status. A number of clinical studies have examined the therapeutic potential of CoQ10 supplementation in the treatment of PD.

In a phase II clinical trial conducted by Schults et al. [49], oral CoQ10 supplementation was found to reduce the functional decline of patients with early-stage PD. In this study, patients were randomly assigned to a placebo or to a CoQ10 treatment group (300, 600 or 1200 mg/day). Disease progression was then determined using the Unified Parkinson’s Disease Rating Scale (UPDRS) at one, four, eight, twelve and sixteen months. The UPDRS assesses the mental and motor capacity of patients, as well as their ability to complete daily living activities. Following eight months of treatment with CoQ10 (300 and 600 mg/d), the mean total UPDRS score of patients was similar. However, it was lower than that of the placebo group, suggesting that CoQ10 supplementation was in some way slowing the disease progression of PD. However, the UPDRS scores of PD patients receiving 1200 mg/d of CoQ10 were not significantly lower than the 300 and 600 mg/d CoQ10 treatment groups, suggesting that there may be a dosage threshold for the gastrointestinal absorption (GI) of CoQ10 into the circulatory system. The efficiency of absorption of CoQ10 formulations has been reported to decrease as the dosage increases, with a suggested GI absorptional block above 2400 mg [50]. A subsequent phase III clinical trial involving six hundred patients was undertaken with PD patients receiving CoQ10 dosages of 1200 or 2400 mg/d [51]. Despite 1200 mg/d being the highest dosage used in the previous study, the mean change in UPDRS score of treated patients was not found to be significantly lower than that of the placebo group, and the researchers concluded that since CoQ10 appeared to show no apparent clinical benefit, they could not recommend its use in the treatment of early-stage PD. The contrasting findings of the clinical studies by Shults et al. [49] and Beal et al. [51] may reflect the broad range of sporadic PD patients used in the two clinical trials, with the heterogeneous patient populations contributing to their contradictory findings. Furthermore, no assessment of an underlying CoQ10 deficiency was determined in the PD patients prior to commencing CoQ10 supplementation in the study by Beal et al. [51], which may explain the limited therapeutic potential of CoQ10 reported.

**Amyotrophic Lateral Sclerosis:** Amyotrophic lateral sclerosis (ALS), otherwise known as motor neuron disease, is a progressive disorder characterised by the degeneration of upper and lower motor neurons within the brain and spinal cord, resulting in the loss of muscle control. Most cases of ALS are sporadic, although there is a familial form, with a typical age of onset at 60 years or 50 years, respectively. A hallmark of ALS is the development of ubiquinated protein aggregates within the motor neurons, resulting in the degeneration of the latter. Although the cause of aggregate formation is not completely understood, mitochondrial dysfunction and oxidative stress have been implicated [52,53]. Although supplemental CoQ10 or its synthetic analogue, MitoQ, prolonged survival in a mouse model of ALS [54,55], a Phase II trial supplementing 2700 mg CoQ10/day for 9 months in 185 ALS patients found insufficient benefits to warrant a Phase III study [56].

**Stroke:** Stroke, by definition, is a disorder in which the normal blood supply to the brain is disrupted; this may result from blockage of a blood vessel by a blood clot (occlusive stroke) or as a result of a blood vessel rupture (haemorrhagic stroke). Mitochondrial dysfunction and oxidative stress have been regarded as hallmarks of ischemia/reperfusion-induced neuronal death following stroke [57,58]. In this regard, Simani et al. [59] found serum CoQ10 levels to be significantly depleted in patients following acute stroke and to correlate with clinical neurological outcomes. However, a randomised controlled study supplementing CoQ10 in acute stroke patients comprised too few patients (22 CoQ10, 22 placebo) and too low a dosage regime (300 mg/day for 4 weeks) to make a definitive conclusion re efficacy [60].

**Multiple system atrophy:** Multiple system atrophy (MSA) is an example of one of the less common neurological disorders, with an age of onset typically in the range of 50–60 years. This disorder results from progressive degeneration of neurons and glia, with subsequent dysfunction of the autonomic nervous system. MSA has been included in the present review since its pathogenesis has been linked to the dysfunction of an enzyme (COQ2; 4-parahydroxybenzoate:polyphenyltransferase) in the CoQ10 synthetic pathway. Several studies have reported a reduction in plasma or post-mortem brain tissue. Thus, in a series of 44 MSA patients, Mitsui et al. [61] found a significant reduction in the mean plasma CoQ10 level of approximately 30% compared to controls. Barca et al. [62] found CoQ10 levels to be significantly depleted (by 40%) in post-mortem cerebellar tissue from MSA patients, compared to controls. In addition, in a study using induced pluripotent stem cell (iPSC)-derived neurons, CoQ10 levels were significantly reduced in MSA patients, particularly those with COQ2 functional variants [63]. To date, there have been no randomised controlled trials of CoQ10 in MSA.

**Ophthalmic disorders:** Age-related disorders of the eye include glaucoma, macular degeneration and cataracts. The eye is exposed to high levels of UV radiation and its associated free radical-induced oxidative stress; in addition, the retina is one of the body’s most metabolically active tissues, with concomitant requirements for energy production and antioxidant protection. Thus, all of the above factors are of relevance to the metabolic roles of CoQ10 and its potential in the treatment of these age-related eye disorders. Glaucoma is a common disorder, which can result in loss of vision if not treated sufficiently early. Glaucoma results from damage to the optic nerve, typically (but not always) resulting from an increase in intraocular pressure, in turn resulting from fluid accumulation. Glaucoma can occur at any age but is most common in individuals aged over 70 years. A number of factors may contribute towards the degeneration of the optic nerve, including mitochondrial dysfunction and subsequent oxidative stress in retinal ganglion cells [64], suggesting a potential role for supplemental CoQ10 in the treatment of this disorder. Thus Qu et al. [65] found CoQ10 levels in retina and choroid from younger (<30 years) and older (>80 years) decreased by approximately 40% with age. The method of CoQ10 administration to the eye then needs to be addressed. Topically applied CoQ10 has poor intraocular penetration and subsequent bioavailability, partly because of its molecular characteristics and partly through the action of the P-glycoprotein (P-gp) efflux pump present in the corneal epithelial cells, which extrudes CoQ10 out of cells. However, increased corneal penetration and intraocular absorption of CoQ10 can be achieved by topical co-administration of CoQ10 with alpha-tocopherol, a known inhibitor of the P-glycoprotein pump [66]. Macular degeneration is a disorder characterised by the loss of vision in the central part of the visual field, again linked to mitochondrial dysfunction and oxidative stress in affected retinal pigment epithelial cells. Macular degeneration typically develops in individuals aged 60 years and above. In a randomised controlled trial, patients with macular degeneration were treated with a combination of CoQ10, acetyl-L-carnitine and n-3 fatty acids over a twelve-month period [67]. Measured parameters of visual function included visual field mean defect, visual acuity (Snellen chart and ETDRS chart), foveal sensitivity as measured by perimetry and fundus alterations as evaluated according to the criteria of the International Classification and Grading System for AMD; at the end of the study period, all four of these parameters showed significant improvement in treated patients compared to placebo.

Cataracts result in a loss of vision caused by the development of opacity in the lens, in turn resulting from denaturation of crystallin protein components of the lens induced by oxidative stress. Cataracts typically develop in individuals aged 50 years and above, affecting more than 50% of people aged 80 years. Kernt et al. [68] showed that the incubation of human lens epithelial cells with CoQ10 significantly reduced light-induced cell damage. A combination of CoQ10 and alpha-tocopherol has been used to reduce post-operable corneal damage after cataract surgery [69,70].

## 5. Why Have Clinical Trials of CoQ10 in Neurological Disorders Been Disappointing?

Studies using animal model systems of a number of neurodegenerative disorders have shown promising evidence of benefit following supplementation with CoQ10. Thus, the administration of CoQ10 has been shown to be beneficial in paraquat-induced or MPTP-induced murine models of Parkinson’s disease by improving behaviour, reducing oxidative stress or preventing loss of dopamine [71,72,73]. Similarly, supplementary CoQ10 reduced oxidative stress, beta-amyloid deposition and cognitive performance in transgenic mouse models of Alzheimer’s disease [74]. In addition, supplementary CoQ10 significantly improved life span in a transgenic mouse model of familial ALS [54]. It is also of note that supplemental CoQ10 prevented the decline in mitochondrial function associated with normal ageing in mice [75].

Mitochondrial dysfunction and oxidative stress have been implicated in the pathogenesis of Parkinson’s disease [76], Alzheimer’s disease [77], ALS [78] and stroke [79]. In clinical studies, CoQ10 levels have been found to be depleted in both the blood and brain cortex tissue of Parkinson’s disease patients [23]. Depleted levels of CoQ10 in blood are associated with an increased risk of developing Alzheimer’s disease [32]. High levels of oxidised CoQ10 (associated with increased oxidative stress) were reported in patients with ALS [80].

On the basis of the cellular functions of CoQ10 and the results of the various studies outlined above, there is, therefore, a clear rationale for clinical studies supplementing CoQ10 in age-related neurodegenerative disorders. However, the outcomes of clinical trials supplementing CoQ10 in various neurological disorders, most notably Alzheimer’s disease, Parkinson’s disease and ALS, have been surprisingly disappointing. Thus, phase II or Phase III trials of high-dose CoQ10 in Alzheimer’s disease (1200 mg/day for 16 weeks), Parkinson’s disease (1200–2400 mg/day for 16 months) or ALS (2700 mg/day for 9 months) failed to slow the progression of these disorders, respectively [35,51,56]. A randomised controlled study supplementing CoQ10 in acute stroke patients comprised too few patients (22 CoQ10, 22 placebo) and too low a dosage regime (300 mg/day for 4 weeks) to make a definitive conclusion re efficacy [60]. In rationalising the outcomes from the above studies, a number of issues need to be considered.

Firstly, was the supplementary CoQ10 sufficiently absorbed from the digestive tract into the bloodstream. Because of the extreme hydrophobicity of the CoQ10 molecule, orally administered CoQ10 has a low bioavailability. Briefly, following transit through the stomach, CoQ10 is subject to micellisation within the duodenum, which facilitates the transport of CoQ10 to the intestinal villi prior to its absorption by enterocytes. CoQ10 is absorbed into enterocytes via a process of passive facilitated diffusion; the facilitator molecule for CoQ10 has not been definitely characterised, but the cholesterol transporter NPC1L1 (Niemann-Pick C1 Like 1) has been suggested as a potential candidate [81,82]. CoQ10 is then incorporated into chylomicrons, which are released into the distal abdominal lymph duct, where they can then enter the systemic blood circulation via the subclavian vein. Chylomicrons in the circulation are taken up by the liver where CoQ10 is then re-packaged into lipoprotein particles, principally, LDL (low density lipoprotein) and VLDL (very low-density lipoprotein) cholesterol, with a relatively small amount of CoQ10 being associated with HDL (high-density lipoprotein) cholesterol [83]. Estimates of exogenous CoQ10 bioavailability following the above process have typically been in the order of 1–5%.

Of the various factors potentially influencing exogenous CoQ10 bioavailability, supplement formulation is a key factor. Thus, the comparative study in human subjects carried out by Lopez-Lluch and colleagues [84] demonstrated the importance of CoQ10 supplement formulations in maximising absorption. The bioavailability of 7 different formulations of CoQ10 (differing by: CoQ10 crystal dispersion status, type of carrier oil, the composition of other excipients and CoQ10 oxidation state) were administered to 14 healthy individuals in a single 100 mg dose using a crossover/washout protocol [83]. The bioavailability of the different formulations was quantified as AUC (area under the curve) following 48 h after administration. A salient point to note from this study is the difference in bioavailability between samples 01 and 02. Both samples contained 100 mg CoQ10 in identical ubiquinone form in a soy carrier oil with similar excipient content and capsule specification. Sample 01 (Bio-Quinone, Pharma Nord) had been subjected to a patented thermal crystal dispersion process, whilst sample 02 had not been treated in this manner. The respective mean AUC and Cmax (concentration maximum) values in the Lopez-Lluch study were 28.0 mg/L/48 h and 1.07 mg/L for sample 01, and 6.89 mg/L/48 h and 0.33 mg/L for sample 2, respectively. The failure to subject crystalline CoQ10 to crystal dispersion, therefore, reduced its bioavailability by approximately 75%. The second point of note relates to the relative bioavailability of the ubiquinone and ubiquinol forms of CoQ10. For the ubiquinol form (sample 05), the AUC and Cmax values were 14.8 mg/L/48 h and 0.49 mg/L, respectively. Thus, the AUC for CoQ10 in ubiquinol form was approximately twice that for ubiquinone form, which had not been subjected to thermal crystal dispersion (sample 02), but was only 52% of that for ubiquinone that had been subject to thermal crystal dispersion (sample 01). These data, therefore, demonstrate:

(i) The importance of CoQ10 crystal dispersion since the failure to disperse CoQ10 crystals to single molecules reduces the bioavailability of CoQ10 by approximately 75%;
(ii) That the relative bioavailability of ubiquinone and ubiquinol forms of CoQ10 depends on its CoQ10 crystal dispersion status and carrier oil/excipient composition.

The above analysis is reproduced from the paper by Mantle and Dybring [83]. 

Thus, in clinical studies, the extent of supplement absorption should always be quantified via one of the standard HPLC-based methods [85]. Normal plasma levels of CoQ10 are in the range 0.5–1.5 mcg/mL [86]. In the Phase III clinical trial of CoQ10 in Parkinson’s disease, the administration of 1200 mg/day CoQ10 raised the mean plasma level to 5.80 mcg/mL after 16 months, whilst a dose of 2400 mg/day raised the mean plasma CoQ10 levels to 9.94 mcg/mL [51]. In the Phase II trial of CoQ10 in ALS, supplementation with CoQ10 at 1800 mg/day raised the mean plasma level to 4.66 mcg/mL after 9 months, and a dose of 2700 mg/day raised the mean plasma CoQ10 level to 5.96 mcg/mL [56]. In the above studies in Parkinson’s disease or ALS, it is, therefore, evident that supplementary CoQ10 substantially raises circulatory levels of CoQ10. However, in a small-scale randomised control trial of CoQ10 (1200 mg/day for 16 weeks) in Alzheimer’s disease, which provided no cognitive benefit, changes in blood CoQ10 levels following supplementation were not measured [35].

The second issue to consider is whether the supplementary CoQ10 was able to cross the blood–brain barrier, and thirdly, how the CoQ10 was distributed between and within brain cells. The latter issues remain largely unknown quantities in human subjects; whilst there is evidence that supplementary CoQ10 can penetrate the blood–brain barrier in animal species, this has yet to be established in humans [87]. In this regard, synthetic analogues of CoQ10, such as idebenone or mitoquinone, have been developed with the intention of improving blood–brain barrier penetration or mitochondria-specific targeting, although the efficacy and safety of such compounds have yet to be fully established in clinical studies [88]. A key issue, therefore, is to determine how CoQ10 may access the blood–brain barrier in humans. In this regard, a recent study by Wainwright et al. [89] using a model system based on porcine brain endothelial cells identified lipoprotein-associated CoQ10 transcytosis in both directions across the in vitro BBB. The uptake of CoQ1 the via the SR-B1 (Scavenger Receptor) and the RAGE (Receptor for Advanced Glycation End products) receptors was comparable to the efflux of CoQ10 via the LDLR (Low-Density Lipoprotein Receptor) transporter, which resulted in no “net” transport of CoQ10 across the BBB. When a CoQ10 deficiency was induced in the model (using p-aminobenzoic acid) treatment, the tight junctions of the BBB were disrupted, and CoQ10 “net” transport to the brain side was increased. Within this area of research, a study by Park et al. [90] is of particular note. In this study, using a rat model of Parkinson’s disease, continuous intrastriatal delivery of low-dose CoQ10 (some four orders of magnitude lower than orally administered CoQ10) showed significant benefits in terms of dopaminergic neuronal loss, as well as behavioural benefits. Whether such an invasive regime could be used for the treatment of Parkinson’s patients is currently an unresolved question. To date, only two randomised controlled trials involving intrastriatal intervention (with agents other than CoQ10) in Parkinson’s disease patients have been reported, both of which involved surgical transplantation, and both of which provided no significant symptomatic benefit [91,92].

With regard to the transport of substances into the brain, a point to consider relates to a difference in dosing regimens between the Phase II and Phase III trials of CoQ10 in Parkinson’s disease. In the Phase II study, CoQ10 in daily doses of 300, 600 or 1200 mg were administered to Parkinson’s disease patients, resulting in a significant slowing of functional decline [49]. In the Phase III study, daily doses of CoQ10 (1200 or 2400 mg) were administered to Parkinson’s disease patients, together with a daily dose of 1200 IU of vitamin E; there was no evidence of significant symptomatic benefit, in contrast to the outcome of the Phase II study [51]. As with the transport of CoQ10 into the brain, the transport of vitamin E into the brain is also poorly understood [93]; the question, therefore, arises as to whether co-administration of a high dose of vitamin E could have inhibited access to the brain for CoQ10, for example via competition for shared lipoprotein or other carrier types.

With regard to the intracellular transport of CoQ10, this process, whether in brain tissue or in other tissues, is currently not well understood. Within cells, a transport mechanism must exist to facilitate the transport of CoQ10 between the subcellular organelles where endogenous CoQ10 is synthesised and those where it is utilised, as well as the subcellular distribution of exogenous CoQ10. Several such mechanisms have been proposed, including Golgi-derived vesicular transport in plant tissues [94] and the transport of CoQ10 via the protein saposin B in human tissues [95].

Patient selection is another potential confounding factor re bioavailability. For example, it is known that Parkinson’s disease is etiologically heterogeneous; thus, mutations in the PINK1 gene (a known cause of Parkinson’s disease) affect the ability of mitochondria to utilise CoQ10 in the energy generation process, so it follows that supplementing with CoQ10 would not benefit this type of Parkinson’s disease patients. In this regard, clinical trials comprising genetically stratified subgroups of Parkinson’s disease patients who might benefit from supplementation with CoQ10 have been proposed by Prasuhn et al. [96], and this approach may lead to more successful outcomes using this type of therapeutic strategy. In addition, the comparative bioavailability clinical study carried out by Lopez-Lluch and colleagues [84] demonstrated the wide inter-individual ability of subjects to absorb a given formulation of CoQ10; in particular that there may be groups of patients with restricted ability to absorb this compound irrespective of formulation type.

Finally, it is useful to compare the outcome of supplementing CoQ10 in the above disorders with a neurological disorder in which CoQ10 supplementation has been clinically successful, namely cerebellar ataxia. Cerebellar ataxia is an autosomal recessive disorder resulting from a primary CoQ10 deficiency (resulting from genetic defects in the CoQ10 biosynthetic pathway). This condition typically manifests in childhood or early adulthood [97]. The early identification of a CoQ10 deficiency in patients with cerebellar ataxia is very important, as patients can show remarkable clinical improvement following CoQ10 supplementation when it is administered at an early stage of the disease. This is illustrated by the clinical studies of Musumeci et al. [98] and Lamperti et al. [99], which reported a significant improvement in the cerebellar function in children or young adults following supplementation with CoQ10 (300–3000 mg/day). The successful outcome of such studies implies that supplemental CoQ10 was able to cross the blood–brain barrier in human subjects. The success or otherwise of CoQ10 supplementation in neurological disorders may, therefore, depend on whether the condition is a primary or secondary deficiency and the stage of disease at which supplementation is attempted. With regard to the safety of CoQ10 supplementation, CoQ10 is generally well tolerated, with no serious adverse events reported in long term use. Furthermore, there are no known toxic effects associated with CoQ10 supplementation, and CoQ10 cannot be overdosed. However, very rarely, individuals may experience mild gastrointestinal disturbance following CoQ10 supplementation, although this does not appear to be dose-related [100].

## 6. Summary

(i) Brain tissue is subject to characteristic changes associated with the ageing process. There is an increased risk of developing particular neurological disorders with increasing age, notably Parkinson’s disease, Alzheimer’s disease, ALS and stroke.

(ii) There is a considerable body of evidence in the published literature implicating mitochondrial dysfunction and oxidative stress in the pathogenesis of the above disorders. There is, therefore, a rationale for the involvement of CoQ10 in the pathogenic mechanism underlying these disorders, given the key role played by CoQ10 in normal mitochondrial function and its role as a major endogenous antioxidant.

(iii) Although a relatively small amount of CoQ10 is obtained from the normal diet, most of the body’s daily CoQ10 requirement is derived from endogenous synthesis, which takes place in most tissues. As people age, the capacity of the body to produce CoQ10 declines, particularly over the age of 50 years. The decline in CoQ10 synthetic capacity, therefore, mirrors the increased risk of developing the neurological disorders outlined above.

(iv) Although studies supplementing CoQ10 in animal models of the above disorders showed significant symptomatic benefit, clinical studies supplementing CoQ10 have been surprisingly disappointing in outcome, most notably in patients with Parkinson’s disease or ALS.

(v) The question, therefore, arises as to why such clinical studies have been unsuccessful. A number of factors may be involved, but particularly whether CoQ10 is able to cross the blood–brain barrier; although this has been demonstrated in brain tissue from several animal species, this has yet to be confirmed in humans.

(vi) Whether exogenous CoQ10 can cross the blood–brain barrier in humans, therefore, remains an outstanding issue and area for future research; in particular, whether the binding of CoQ10 to LDL/VLDL carriers is a necessary requirement for CoQ10 to access the blood–brain barrier.

(vii) Another relevant issue requiring further research is the mechanism(s) by which exogenous CoQ10 is distributed within brain tissue cells once it has crossed the blood–brain barrier; this is also the case for endogenously synthesised CoQ10 and for tissues other than brain tissue.
